# Should an intercalated degree be compulsory for undergraduate medical students?

**DOI:** 10.3402/meo.v20.29392

**Published:** 2015-10-01

**Authors:** Aaron B. Philip, Sunila J. Prasad, Ankur Patel

**Affiliations:** School of Medicine, Faculty of Medicine, Imperial College London, London, United Kingdom

**Keywords:** iBSc, mandatory, medical school, research, projects, academic, BMedSci

## Abstract

Undertaking an intercalated year whilst at medical school involves taking time out of the medicine undergraduate programme in order to pursue a separate but related degree. It is widely seen as a challenging but rewarding experience, with much to be gained from the independent project or research component of most additional degrees. However, whilst intercalating is encouraged at many universities and is incorporated into some undergraduate curricula, it is by no means compulsory for all students. The literature would suggest that those who have intercalated tend to do better academically, both for the remainder of medical school and after graduating. Despite this, the issue of making intercalation mandatory is one of considerable debate, with counter-arguments ranging from the detrimental effect time taken out of the course can have to the lack of options available to cater for all students. Nonetheless, the research skills developed during an intercalated year are invaluable and help students prepare for taking a critical evidence-based approach to medicine. If intercalated degrees were made compulsory for undergraduates, it would be a step in the right direction. It would mean the doctors of tomorrow would be better equipped to practise medicine in disciplines that are constantly evolving.

For those for whom medicine is their first degree, the novel stimuli and the breadth of knowledge required can be overwhelming. Whilst at medical school, universities aim to address this by instilling fundamental principles and by creating a firm foundation to prepare students for their future careers. However, whilst this might provide students with the core proficiencies necessary, it is often difficult to teach some of the independent research skills essential for critically analysing evidence in practise. These skills are often best developed practically by undertaking projects under the supervision of an experienced researcher and by continual exposure to the scientific method. It is within this context that the issue of intercalation can be highlighted. Intercalation is defined as suspending the medical degree in order to pursue a related topic, usually for a year, followed by a subsequent return to the original course. Typically, this year out of the undergraduate degree would involve an allocated period of time devoted to a project or independent research. The intercalated Bachelor of Science (iBSc) degree is one of the most common forms in which intercalation exists for medical students and has traditionally been held in high regard. This is especially true in the United Kingdom (UK) where several medical schools already incorporate an iBSc or a Bachelor of Arts (BA) as a compulsory part of their undergraduate course, while the University of Nottingham integrates a similar BMedSci degree into their 5-year programme. However, whilst intercalating is often encouraged by most medical schools, it is by no means obligatory. As such, we would like to discuss whether an iBSc should be mandatory for all undergraduate medical students.

The literature suggests that students who undertake an iBSc are better prepared for the rigours of academia and have an increased interest in pursuing research in their future careers (1–3). Furthermore, it is reported that students who intercalate develop better deep and strategic learning skills compared with those who do not take a year out (2). Student opinion also reveals a largely positive attitude towards iBScs, with those completing an additional degree showing a high level of satisfaction (1, 3, 4).

Moreover, the skills developed during a year out from the undergraduate course are both advantageous and practical; this was demonstrated in a study by Devi et al., which found that a mentored student project helped to improve students’ research skills in a medical school in India (5). The project component of nearly all intercalated degrees incorporate some form of original research or literature review, and it is this process that fosters both a curiosity for innovation and an appreciation for the scientific method.

The benefits of an intercalated degree might also extend to future academic performance while still at medical school. Certain studies exploring the effects that an intercalated degree can have on subsequent exam performance have found that students with an iBSc perform better in their examinations in later years compared with those who do not undertake an additional degree (6, 7). However, not all research has been able to demonstrate this tangible benefit, with one group finding that an iBSc had little effect on subsequent exam results in students’ first clinical year (8). Interestingly, the authors of this study hypothesised that this lack of significant results might be due to over-adjustments made to account for the finding that students who intercalated earlier had better examination results in their previous years (8).

It must also be considered that as the proportion of students intercalating increases, some of the original benefits may be lost, perhaps due to resources being spread too thinly (2). Indeed, a study at Bristol and Sheffield Medical Schools identified that having a clinical academic supervisor had a positive impact on the number of publications and poster presentations that students obtained (4). This would suggest that if intercalated degrees were to be made compulsory, efforts would need to be made to ensure that adequate resources were available and that there were enough staff well-suited to the task of supervising student projects.

A potential counter-argument to implementing a mandatory iBSc is that not all medical students would be in favour of interrupting their studies. This could be because they may not want to complete a degree in a potentially obscure topic, or add another year to their course (9). Some would also argue that the gap between clinical years can be a stumbling block, along with the increased financial burden caused by a delay in entering paid employment, especially with the recent rise in tuition fees for students in the UK (4). Nonetheless, the role evidence-based medicine has in the future of healthcare as a whole is undeniably important. Investing a year in an iBSc, where one would accrue the skills necessary for taking a critical evidence-based approach, is clearly not a wasted venture and is highly likely to pay dividends for many years to come.

Another reason why some might object to making an iBSc compulsory is the limited clinical relevance that certain degrees provide for individuals who are not particularly interested in academia. The lack of subject options that are targeted at those considering a career in primary care is a further concern. However, over the past few years the range of subjects offered to students has risen dramatically, with a variety of degree options ranging from medical specialties to broader topics such as global health and the humanities. Furthermore, the choice to intercalate at another university significantly increases the range of opportunities available to students. It is not clear whether the benefits of an intercalated degree apply to all possible courses, but undoubtedly the research skills necessary for the project component of nearly all iBScs would be transferrable when critically analysing evidence for use in practise.

On balance, a compulsory iBSc for medical students would result in more students reaching their academic potential, as well as developing the skills needed to practise medicine in disciplines that are dynamic and progressive. We strongly believe that a year out of the undergraduate course would help to craft a more robust workforce, ready to tackle the problems of tomorrow.
